# Effect of mode of hydrocortisone administration on glycemic control in patients with septic shock: a prospective randomized trial

**DOI:** 10.1186/cc5696

**Published:** 2007-02-16

**Authors:** Pekka Loisa, Ilkka Parviainen, Jyrki Tenhunen, Seppo Hovilehto, Esko Ruokonen

**Affiliations:** 1 Department of Intensive Care, Päijät-Häme Central Hospital, Keskussairaalankatu 7, FI 15850 Lahti, Finland; 2 Department of Intensive Care, Kuopio University Hospital, P.O. Box 1777, FI 70211 Kuopio, Finland; 3 Department of Intensive Care, Tampere University Hospital, P.O. Box 2000, FI 33521 Tampere, Finland; 4 Department of Intensive Care, South Carelian Central Hospital, Valto Käkelän katu 1, FI 53130 Lappeenranta, Finland

## Abstract

**Introduction:**

Low-dose hydrocortisone treatment is widely accepted therapy for the treatment of vasopressor-dependent septic shock. The question of whether corticosteroids should be given to septic shock patients by continuous or by bolus infusion is still unanswered. Hydrocortisone induces hyperglycemia and it is possible that continuous hydrocortisone infusion would reduce the fluctuations in blood glucose levels and that tight blood glucose control could be better achieved with this approach.

**Methods:**

In this prospective randomized study, we compared the blood glucose profiles, insulin requirements, amount of nursing workload needed, and shock reversal in 48 septic shock patients who received hydrocortisone treatment either by bolus or by continuous infusion with equivalent dose (200 mg/day). Duration of hydrocortisone treatment was five days.

**Results:**

The mean blood glucose levels were similar in the two groups, but the number of hyperglycemic episodes was significantly higher in those patients who received bolus therapy (15.7 ± 8.5 versus 10.5 ± 8.6 episodes per patient, *p *= 0.039). Also, more changes in insulin infusion rate were needed to maintain strict normoglycemia in the bolus group (4.7 ± 2.2 versus 3.4 ± 1.9 adjustments per patient per day, *p *= 0.038). Hypoglycemic episodes were rare in both groups. No difference was seen in shock reversal.

**Conclusion:**

Strict normoglycemia is more easily achieved if the hydrocortisone therapy is given to septic shock patients by continuous infusion. This approach also reduces nursing workload needed to maintain tight blood glucose control.

Trial Registration Number

ISRCTN98820688

## Introduction

Surviving sepsis guidelines recommend low-dose corticosteroids for the treatment of vasopressor-dependent septic shock [1]. In prospective randomized trials, hydrocortisone therapy has shown a significant effect in septic shock reversal [2,3], and in one large prospective study, a treatment with a hydrocortisone-fludrocortisone combination also reduced mortality in the subgroup of patients who had a poor adrenal response in the adrenocorticotrophic hormone stimulation test [4]. The recommended hydrocortisone dosage is 200 to 300 mg daily in three or four divided doses. Alternatively, hydrocortisone may be administered by continuous infusion [5].

The tolerability of hydrocortisone therapy has been good in previous studies and there is no evidence that corticosteroids have increased the risk of gastrointestinal bleeding or risk of secondary infections when used in septic shock [4,6]. However, hydrocortisone is a potent glucocorticoid and it stimulates gluconeogenesis in both liver and peripheral tissues. It is possible that corticosteroid treatment may induce hyperglycemia and that the frequency of insulin use may increase with corticosteroid exposure [7]. These effects must also be considered as major adverse events in critically ill patients. Impaired glycemic control has been associated with increased mortality in a heterogeneous population of critically ill patients [8], and van den Berghe and coworkers [9] showed that preventing hyperglycemia with insulin substantially improved outcome in critically ill surgical patients. This survival benefit was also observed in a recent prospective study in a medical intensive care unit (ICU) population that required ICU treatment for more than three days [10]. In addition, corticosteroids may increase the risk of critical illness polyneuropathy and myopathy, and therefore the use of corticosteroids may be associated with protracted weaning from mechanical ventilation [7,11]. Prolonged hyperglycemia is one possible pathophysiologic mechanism behind these complications [12].

So far, no studies have compared bolus versus continuous hydrocortisone infusion regimen and their effects to blood glucose profiles in septic shock [2,13]. International guidelines do not precisely indicate which treatment modality would be better [5]. The hypothesis of this study was that continuous hydrocortisone infusion would reduce the occurrence of hyperglycemic and hypoglycemic episodes when compared to conventional bolus treatment. The purposes of this study were to investigate how the different corticosteroid treatment modalities would influence glucose profiles in septic shock and to compare the reversal of shock and nursing workload needed between two different hydrocortisone regimens.

## Materials and methods

This prospective study was conducted between July 2005 and April 2006 in the ICUs of Kuopio University Hospital (Kuopio, Finland), Tampere University Hospital (Tampere, Finland), South Carelian Central Hospital (Lappeenranta, Finland), and Päijät-Häme Central Hospital (Lahti, Finland). The study protocol was approved by the local ethics committees, and informed consent was obtained from the patients or their first-degree relatives.

### Patients

Patients were prospectively enrolled in the study if they met the criteria for septic shock defined according to the American College of Chest Physicians/Society of Critical Care Medicine Consensus Conference: (a) the presence of systemic inflammatory response syndrome (manifested by two or more of the following criteria: fever [temperature of more than 38°C] or hypothermia [temperature of less than 35.5°C], tachycardia [more than 90 beats per minute], tachypnea [more than 20 breaths per minute], and leukocytosis or leukopenia [white blood cell count of more than 12,000/mm^3 ^or less than 4,000/mm^3^, respectively]), (b) documented source of infection, and (c) hypotension despite adequate fluid resuscitation (systolic blood pressure of less than 90 mm Hg or a decrease of systolic blood pressure by 40 mm Hg or more from the baseline) [14]. In addition, patients had to receive norepinephrine at any dose to maintain mean arterial blood pressure above 65 mm Hg. Patients under 18 years of age, patients with pre-existing diabetes, and patients receiving glucocorticoids were excluded from the study. Also, patients who died within 24 hours after the randomization were excluded from the analysis. APACHE (Acute Physiology and Chronic Health Evaluation) II score [15] and SAPS (Simplified Acute Physiology Score) II [16] were calculated and the severity of organ dysfunction was assessed using SOFA (Sepsis-related Organ Failure Assessment) score [17] at the time of ICU admission. Hemodynamic variables were recorded with arterial and Swan-Ganz catheters. A pulmonary artery catheter was used in 42 (88%) patients on the basis of clinical judgment.

### Study intervention

When patients were considered to benefit from the corticosteroid treatment, they were randomly assigned to receive hydrocortisone either by a conventional bolus therapy (50-mg bolus of hydrocortisone every six hours intravenously) or by continuous infusion with equivalent dose (200 mg/day). Hydrocortisone treatment was started according to clinical judgment when patients required high-dose or increasing norepinephrine support [18]. Hydrocortisone was given in hydrocortisone sodium succinate (Solu-Cortef^®^; Pharmacia, now part of Pfizer Inc, Täby, Sweden), and when continuous infusion was used, hydrocortisone was diluted in physiologic saline. Randomization was performed in groups of four patients by means of sequentially numbered opaque envelopes. The duration of hydrocortisone treatment was five days.

After the randomization, a maintenance infusion of 5% glucose was started at the rate of 30 ml/kg per day. At the same time, a protocol-based enteral nutrition with standard formulas (1 kcal/ml) was initiated. Enteral feeding was started at 500 ml/day with daily increments of 500 ml if possible. The maximum amount of enteral nutrition was set at 1,500 ml/day. Blood glucose levels were monitored from the arterial line every two hours during the study period, and the goal was to maintain blood glucose levels between 4 and 7 mmol/l (72 to 126 mg/dl). Blood glucose measurements were performed with an arterial blood gas analyzer. When the blood glucose level exceeded 7 mmol/l (126 mg/dl), an insulin infusion of 1 IU/ml (Actrapid^®^; Novo Nordisk A/S, Bagsvaerd, Denmark) was started and the dose was adjusted according to a strict algorithm (Table  1).

**Table 1:** Algorithm for glucose control in the study

Initial infusion			
Blood glucose		Insulin infusion rate	Control interval

(mmol/l)	(mg/dl)		(hours)

7.0–9.9	126–179	1 IU/hour	2
10–11.9	180–214	2 IU/hour	1–2
> 12	> 215	4 IU/hour	1–2

Maintenance infusion			

Blood glucose		Insulin infusion rate	Control interval

(mmol/l)	(mg/dl)		(hours)

< 2.5	< 45	10 % glucose 150 ml iv..	0.5
< 3.0	< 54	Stop insulin	1
3.1–3.9	55–71	Reduce insulin dose by half	1
4.0–4.9	72–89	Reduce by 0.5 IU/hour	2
5.0–6.9	90–125	Insulin dose unchanged	2
7.0–9.9	126–179	Increase by 0.5–1 IU/hour	2
10–11.9	180–214	Increase by 1 IU/hour	2
> 12.0	> 215	Increase by 2 IU/hour	2

i.v., intravenously.

### Sample size and statistical analysis

A sample size was calculated on the basis of detecting a difference of 1 mmol/l in mean blood glucose levels between the study groups. A standard deviation (SD) of 1 mmol/l in blood glucose level was assumed when calculating a sample size based on previous studies [9]. A minimum of 17 patients were required in each group (α = 0.05, power = 80%). ICU mortality was expected to be 30% and therefore 24 patients were randomly assigned in both groups. Results are reported as mean ± SD. Descriptive data were analyzed using the unpaired *t *test for the continuous variables, and the categorical data were analysed using a χ^2 ^test. Blood glucose profiles, insulin requirements, and serial hemodynamic data were compared with the analysis of variance for repeated measurements. Kaplan-Meier curves were calculated for shock reversal, and the comparison between the groups was performed with the log-rank test. All randomly assigned patients were included for mortality and shock reversal analysis, and the patients who died due to refractory hypotension during the study period were considered as not having reversed septic shock. A *p *value of less than 0.05 was considered significant. Statistical analysis was performed using the SPSS 13.0 version (SPSS Inc., Chicago, IL, USA).

The primary endpoint in the study was the difference in the mean blood glucose levels between the study groups and the occurrence of hyper- and hypoglycemic episodes. Secondary endpoints included the reversal of shock and the amount of nursing workload required to maintain strict normoglycemia. The nursing workload was estimated by recording the number of adjustments in insulin infusion during the study period. Hyperglycemia was defined as a blood glucose level of more than 7 mmol/l (126 mg/dl) and severe hyperglycemia as a blood glucose level of more than 8.3 mmol/l (150 mg/dl) [19]. Hypoglycemia was defined as a blood glucose level of less than 3 mmol/l (54 mg/dl) and severe hypoglycemia as a blood glucose level of less than 2.2 mmol/l (40 mg/dl) [9]. Reversal of shock was defined as a stable mean arterial pressure of more than 65 mm Hg for at least 24 hours without norepinephrine support.

## Results

**Figure 1:**
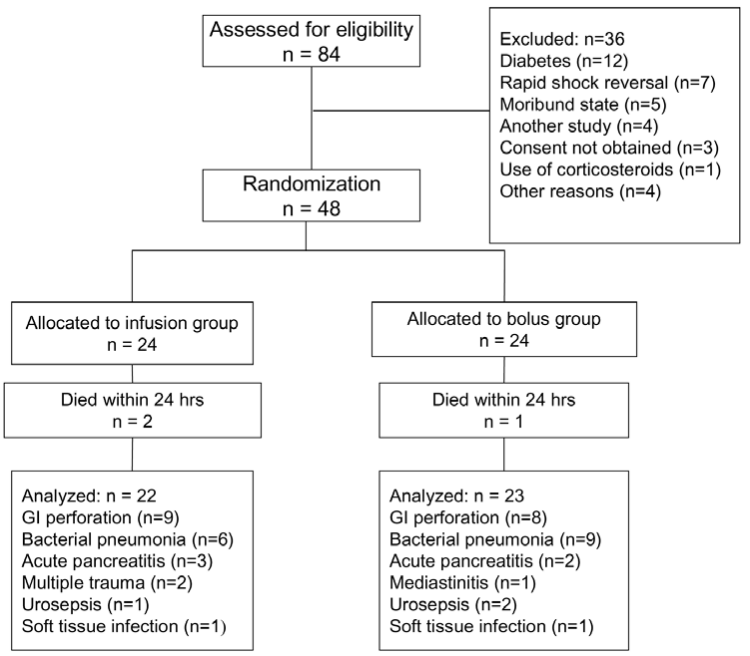
Flow diagram of the study. GI, gastrointestinal.

A total of 48 patients were enrolled in the study. Two patients in the infusion group and one in the bolus group died within 24 hours after the randomization and these patients were excluded from the final analysis (Figure 1). These three patients were included in shock reversal and mortality analysis. Demographic data and the clinical characteristics of the patients are presented in Table 2. There were no differences between the two groups at the beginning of the study. In the infusion group, 68% of the patients (15/22) were surgical patients and the corresponding value in the bolus group was 48% (11/23); this difference was not statistically significant. Five patients (three patients in the bolus group and two patients in the infusion group) underwent surgical procedures during the study. In two patients (one in the infusion group and one in the bolus group), enteral nutrition had to be stopped due to surgery, and in both patients one glucose measurement was missed during the operation period.

**Table 2:** Patient characteristics in the study

	Bolus group (*n *= 23)	Infusion group (*n *= 22)	*p *value
Age (years)	61 ± 17	60 ± 16	0.732
APACHE II score	22.7 ± 5.6	22.5 ± 7.9	0.950
SAPS II	51.5 ± 11.3	52.2 ± 14.6	0.860
SOFA score	10.0 ± 2.1	10.3 ± 2.4	0.707
PaO_2_/FiO_2 _ratio (mm Hg)	193 ± 110	176 ± 75	0.532
Cardiac index (l/minute per m^2^)	3.8 ± 1.7	3.6 ± 1.5	0.555
Mean arterial pressure (mm Hg)	62 ± 7.5	65 ± 7.9	0.188
Systemic vascular resistance (dyn·s/cm^5^)	623 ± 221	731 ± 254	0.158
SvO_2 _(percentage)	61 ± 7.6	64 ± 12	0.462
Hemoglobin (g/l)	106 ± 22	105 ± 19	0.890
Leukocyte count (10^9^/l)	14.1 ± 9.2	9.6 ± 7.7	0.091
Platelet count (10^9^/l)	180 ± 103	141 ± 92	0.191
Plasma C-reactive protein (mg/l)	223 ± 115	197 ± 99	0.428
Serum lactate (mmol/l)	2.3 ± 1.6	2.8 ± 1.9	0.471
Norepinephrine dose (μg/kg per minute)	0.22 ± 0.12	0.19 ± 0.16	0.579
ICU mortality, *n *(percentage)	4 (17%)	7 (29%)	0.494

APACHE II, Acute Physiology and Chronic Health Evaluation II; ICU, intensive care unit;

PaO_2_/FiO_2_, arterial oxygen pressure/inspiratory fractional oxygen content; SAPS II,

Simplified Acute Physiology Score II; SOFA, Sepsis-related Organ Failure Assessment;

SvO_2_, mixed venous oxygen saturation.

**Figure 2:**
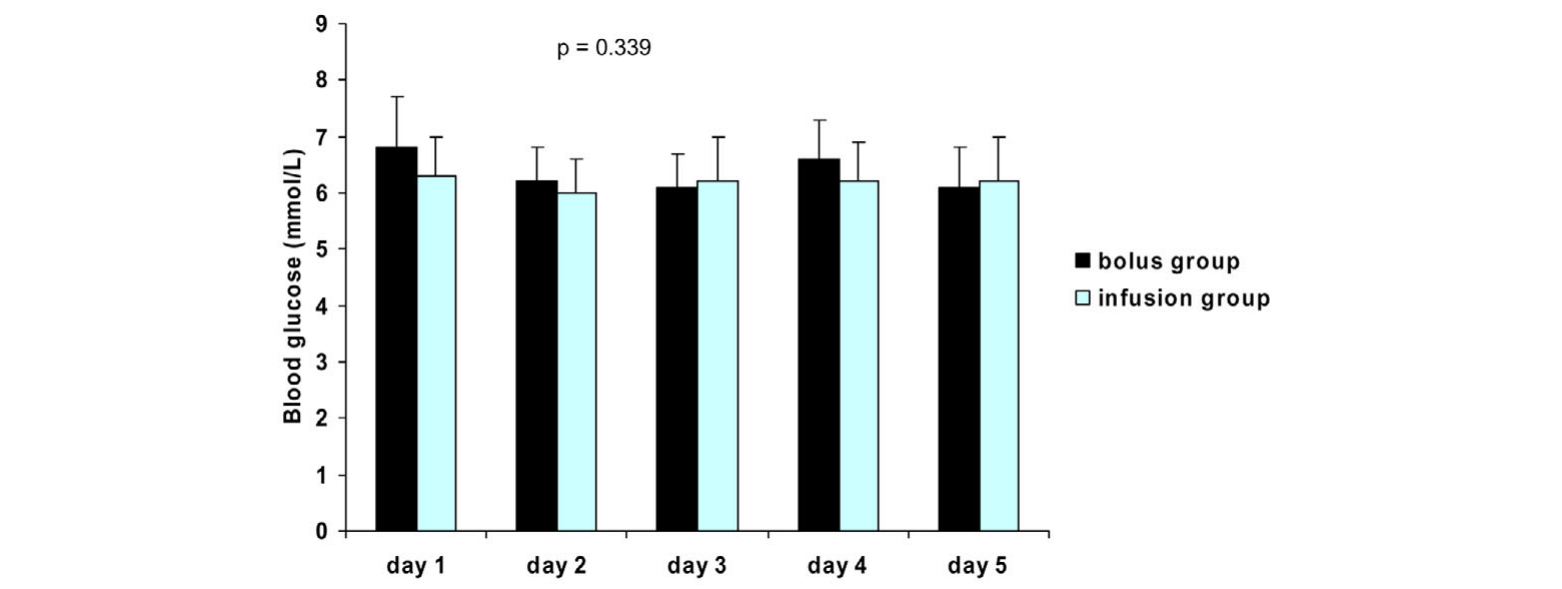
Blood glucose levels (mean ± standard deviation) in the study groups. *P *values represent the difference between the study groups (analysis of variance).

**Figure 3:**
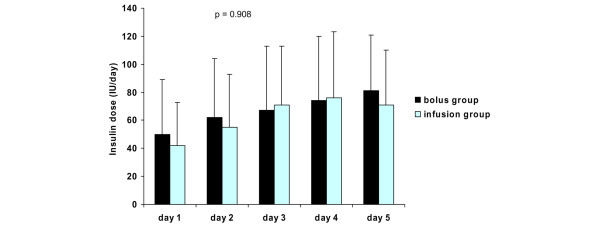
Insulin requirements (mean ± standard deviation) in the study groups. *P *values represent the difference between the study groups (analysis of variance).

**Figure 4:**
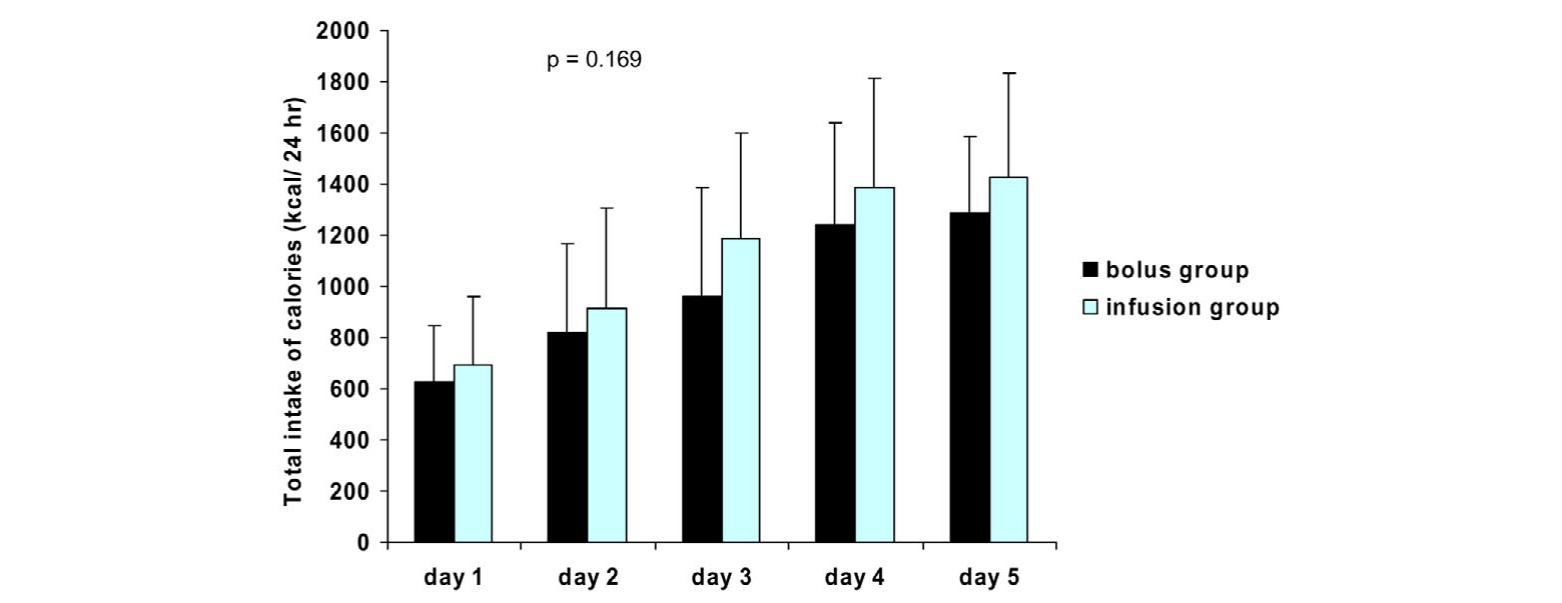
Intake of calories (mean ± standard deviation) in the study groups. *P *values represent the difference between the study groups (analysis of variance).

**Figure 5:**
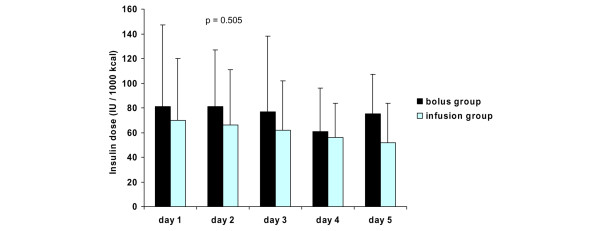
Insulin requirements adjusted to administered calories (mean ± standard deviation) in the study groups. *P *values represent the difference between the study groups (analysis of variance).

The mean daily blood glucose levels, insulin requirements, and intake of calories are presented in Figures 2, 3 and 4, respectively. There were no differences in mean daily blood glucose levels between the study groups. Also, insulin requirements and intake of calories were similar in the two groups. All patients received exogenous insulin during the study. When insulin requirements were adjusted to administered calories, a trend of lower insulin requirements in the infusion group was observed throughout the study period (Figure 5). However, due to large individual variations, the difference between the groups was not statistically significant.

The data concerning glycemic control are presented in Table 3. A total of 2,428 blood glucose measurements were performed during the study, and 1,804 of them (74.3%) were within the predetermined target range. The overall mean blood glucose level was lower in the infusion group, but this difference of 0.2 mmol/l cannot be considered clinically significant. Although the mean blood glucose levels were quite similar, the hyperglycemic (more than 7 mmol/l [126 mg/dl]) episodes were more common in the bolus group than in the infusion group (*p *= 0.039). Severe hyperglycemia (blood glucose of more than 8.3 mmol/l [150 mg/dl]) was rare in both study groups and hypoglycemic episodes also were uncommon. Three hypoglycemic (blood glucose of less than 3 mmol/l [54 mg/dl]) episodes were observed in the bolus group and only one in the infusion group. Severe hypoglycemia (blood glucose of less than 2.2 mmol/l [40 mg/dl]) was not observed in either study group. The amount of nursing workload needed to maintain normoglycemia was higher in the bolus group: more insulin infusion rate adjustments were needed in the bolus group compared to infusion-treated patients (*p *= 0.038).

**Table 3:** Glycemic control in study groups

	Bolus group (*n *= 23)	Infusion group (*n *= 22)	*p *value
Mean blood glucose (mmol/l)	6.4 ± 0.7	6.2 ± 0.7	0.040
Blood glucose variation coefficient (percentage)	20.2 ± 6.9	16.5 ± 4.8	0.063
Blood glucose > 7 mmol/l (episodes per patient)	15.7 ± 8.5	10.5 ± 8.6	0.039
Blood glucose > 8.3 mmol/l (episodes per patient)	3.6 ± 3.4	2.6 ± 3.2	0.383
Mean insulin dose (IU/day)	66 ± 43	61 ± 40	0.381
Insulin infusion adjustments (number per patient per day)	4.7 ± 2.2	3.4 ± 1.9	0.038
Blood glucose < 3 mmol/l (episodes per group)	3 (13%)	1 (4.5%)	0.609
Blood glucose < 2.2 mmol/l (episodes per group)	0	0	n.s.

To convert glucose values to milligrams per deciliter, multiply by 18,015. n.s., not significant.

Serial hemodynamic data are presented in Table 4. The reversal of shock was similar in the study groups. The vasopressor support could be withdrawn within 48 hours in 14 (58%) of the patients in the bolus group, and the corresponding value in the infusion group was 12 (50%). After five days, vasopressors were withdrawn in 20 patients (83%) in the bolus group and in 15 patients (63%) in the infusion group. Four patients in the infusion group and two patients in the bolus group died due to refractory hypotension during the study period. The overall ICU mortality was 23%.

**Table 4:** Hemodynamic parameters in study groups

	Day 1	Day 2	Day 3	Day 4	Day 5	*p *value
Heart rate (beats per minute)						
Bolus group	107 ± 20	93 ± 20	85 ± 20	78 ± 22	86 ± 21	0.93
Infusion group	100 ± 21	95 ± 19	87 ± 23	86 ± 22	83 ± 15	
Mean arterial pressure (mm Hg)						
Bolus group	62 ± 7.5	75 ± 12	79 ± 13	85 ± 15	87 ± 15	0.06
Infusion group	65 ± 7.9	72 ± 9.4	72 ± 12	75 ± 13	79 ± 19	
Cardiac index (liters/minute per m^2^)						
Bolus group	3.8 ± 1.7	3.5 ± 1.1	3.5 ± 0.9	3.4 ± 1.0	3.4 ± 0.7	0.52
Infusion group	3.6 ± 1.5	3.9 ± 1.3	3.5 ± 1.1	3.6 ± 1.3	3.8 ± 1.5	
SvO_2 _(percentage)						
Bolus group	61 ± 7.6	67 ± 7.2	69 ± 7.3	70 ± 7.6	69 ± 7.0	0.50
Infusion group	64 ± 12	65 ± 10	63 ± 11	66 ± 13	70 ± 13	
SVR (dyn·s/cm^5^)						
Bolus group	623 ± 221	770 ± 323	861 ± 249	999 ± 334	1,061 ± 200	
Infusion group	731 ± 254	705 ± 248	818 ± 274	836 ± 332	832 ± 214	
Shock reversal, *n *(percentage)						
Bolus group	0/24 (0%)	3/24 (13%)	14/24 (58%)	18/24 (75%)	20/24 (83%)	0.48
Infusion group	0/24 (0%)	5/24 (21%)	12/24 (50%)	14/24 (58%)	15/24 (63%)	

*P *values represent difference between the study groups (analysis of variance and log-rank test). SvO_2_, mixed venous oxygen saturation; SVR, systemic vascular resistance.

## Discussion

The main findings in the present study were that the hyperglycemic episodes were more common in those patients who received hydrocortisone in bolus therapy and that the amount of nursing workload needed to maintain normoglycemia was higher in bolus-treated patients. Our findings suggest that, in septic shock, strict normoglycemia is more easily achieved with continuous hydrocortisone infusion. However, the differences between the study groups were rather marginal and in both groups the normoglycemic goal could be achieved quite successfully.

The most important risk associated with intensive insulin therapy is the occurrence of severe hypoglycemia. This risk seems especially likely to increase if the target range for the glucose control is set at 4.4 to 6.1 mmol/l (80 to 110 mg/dl). In the study of van den Berghe and coworkers [10], severe hypoglycemic episodes (less than 2.2 mmol/l [40 mg/dl]) were observed in 25% of the long-stay ICU patients, and more importantly, hypoglycemic episodes were associated with increased mortality. In severely ill ICU patients, this risk seems to be higher than in postoperative patients, and patients with sepsis are especially vulnerable to hypoglycemia [10,20,21]. In our study, only four episodes (8.8%) of hypoglycemia (blood glucose of less than 3 mmol/l [54 mg/dl]) were detected, and more importantly, no severe hypoglycemic (less than 2.2 mmol/l [40 mg/dl]) episodes were observed in either study group. These findings suggest that even a slightly more liberal glucose control will prevent dangerous hypoglycemic episodes very effectively. Other authors have also suggested that blood glucose control might be somewhat more liberal than in the study of van den Berghe and coworkers [22].

Certain limitations of this study should be addressed. The study was not placebo-controlled or blinded. The major limitation in our study was that the nutritional support in individual patients was rather heterogeneous despite the unambiguous feeding protocol. The majority of the study patients had septic shock due to gastrointestinal primary disease (gastrointestinal perforation or acute pancreatitis) and in these patients the enteral feeding could not always be increased according to the study protocol. In four patients (two in both groups), parenteral nutrition was initiated during the study because enteral feeding was not possible. In 16 patients, the maximum intake of calories remained below 15 kcal/kg per day, and in eight patients this underfeeding was due to the problems associated with enteral nutrition. Additionally, nutritional goals were not achieved in eight patients because they either died or were discharged to the general ward before the completion of the five day study period. The remaining eight patients received poor nutrition because they either died or were discharged to the general ward before the study period was completed. However, in those patients who stayed in the ICU for the entire study period, the nutritional goals were achieved quite successfully: the mean intake of calories in these patients was 19.0 ± 6.0 kcal/kg per day with no detectable differences between study groups. The standardization of nutritional support has also been difficult in other trials concerning critically ill patients [23].

## Conclusion

Continuous hydrocortisone infusion reduced the number of hyperglycemic episodes, and this approach also reduced the nursing workload during intensive insulin therapy. Strict normoglycemia is more easily achieved if hydrocortisone therapy is given to septic shock patients by continuous infusion.

## Key messages

• In septic shock, continuous hydrocortisone infusion will reduce the number of hyperglycemic episodes during intensive insulin therapy.

• Continuous hydrocortisone infusion will also reduce the nursing workload needed to maintain tight blood glucose control.

## Abbreviations

ICU = intensive care unit; SD = standard deviation.

## Competing interests

The authors declare that they have no financial competing interests (reimbursements, fees, funding, or salary from an organization) that may gain or lose financially from the publication of this manuscript. The authors also declare that they do not hold any stocks or shares that may gain or lose financially from the publication of this manuscript. The authors do not have any non-financial competing interests to declare in relation to this manuscript.

## Authors' contributions

PL participated in the study design and data collection, performed statistical analysis, and wrote the manuscript. IP participated in the study design, data collection, and analysis and interpretation of the results. JT participated in the study design, data collection, and analysis and interpretation of the results and helped to write the manuscript. SH participated in the study design and data collection and contributed to the revision of the manuscript. ER participated in the study design and in the analysis and interpretation of the results and helped to write the manuscript. All authors read and approved the final manuscript.
